# A Case Series of SARS-CoV-2 Omicron Variant in Patients With Acute Leukemia

**DOI:** 10.7759/cureus.25196

**Published:** 2022-05-21

**Authors:** Elrazi A Ali, Mohammed A Alamin, Mohammad Abu-Tineh, Khalid Ahmed, Awni Alshurafa, Waail Rozi, Mohamed A Yassin

**Affiliations:** 1 Internal Medicine, Hamad Medical Corporation, Doha, QAT; 2 Hematology, Hamad Medical Corporation, Doha, QAT; 3 Hematology, Hamad General Hospital, Doha, QAT

**Keywords:** acute myeloid leukemia (aml), acute leukemia, covid-19, omicron variant, sars-cov-2

## Abstract

Coronavirus disease 2019 (COVID-19) is a respiratory viral illness caused by coronavirus 2 (SARS-CoV-2). The disease often presents with non-specific symptoms, including fever, and fatigue, usually associated with respiratory symptoms (eg., cough) and other systemic involvement. The primary strategy to prevent transmission and reduce the disease severity of the SARS-CoV-2 infection is through vaccination. However, the virus had shown significant changes and mutations that resulted in the emergence of different strains. Each strain varies in its virulence, disease severity, and the body's immune system response. Previous reports showed that the Omicron variant causes mild disease. Little is known about the effect of Omicron in patients with acute leukemia. We present three patients with acute leukemia who had an infection with the Omicron variant of the SARS-CoV-2 virus.

## Introduction

Severe acute respiratory syndrome coronavirus 2 (SARS-CoV-2) has quickly expanded worldwide since its first outbreak in Wuhan, China, in 2019, resulting in global epidemics. Patients with comorbidities are prone to develop severe illnesses [1,2]. The SARS-CoV-2 infection manifests primarily as respiratory symptoms, although other vital organs such as the liver, kidney, and skin can also be affected [3-5]. Additionally, patients are at risk of increased thrombosis [6]. Despite the availability of protective vaccines, SARS-CoV-2 spike proteins had several mutations that resulted in the development of novel stains. This resulted in Alpha, Beta, Gamma, Delta, and Omicron variants. Leukemia is a hematological condition in which the production of white blood cells is uncontrolled. The typical presentation of leukemia includes bleeding, infection, or due symptoms of anemia. The effect of SARS-CoV-2 on patients with leukemia is variable. The SARS-CoV-2 disease is frequently associated with an elevated white cell count in patients with chronic lymphocytic leukemia, and mortality is related to age [7,8]. Previous studies showed that acute leukemia with SARS-CoV-2 infection had increased mortality [9]. However, data about the impact of the Omicron variant of the SARS-CoV-2 virus on patients with acute leukemia is small. We report three acute leukemia cases developed infection with Omicron variant of SARS-CoV-2 virus. The series shows the effect of the Omicron variant on this group of patients.

## Case presentation

Case 1

**Figure 1:**
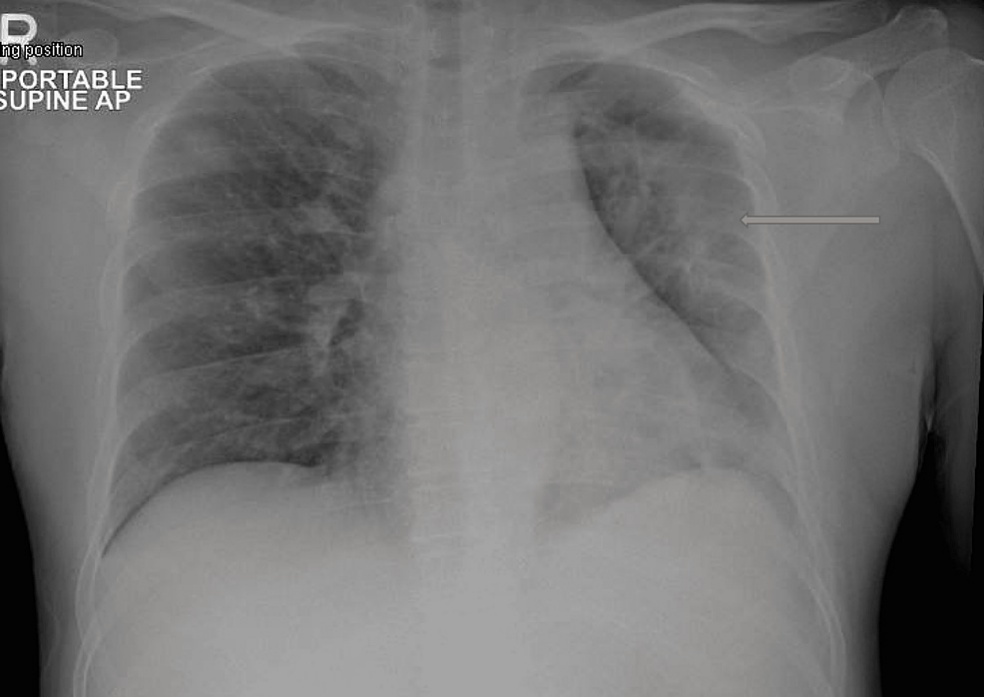
Anteroposterior chest X-ray chest of patient 1 showing reduction of volume of the left lung with scattered patchy consolidations likely pneumonic indicated by the arrow.

A 44-year-old male with acute myeloid leukemia (the acute monocytic type with normal cytogenetics), intermediate-risk, received two induction phases 3+7 (idarubicin and cytarabine) protocol and first consolidation HIDAC protocol, along with intrathecal methotrexate as CNS prophylaxis. He was discharged from the hospital about six weeks after the first consolidation phase. One month after discharge in January 2022, during a routine visit to the hospital for a supportive platelets transfusion (other cell lines were recovered), the patient complained of upper respiratory tract infection symptoms with a sore throat and mild cough. He was found to have a fever of (38 C) and was tested positive for SARS-CoV-2 infection, the Omicron variant by polymerase chain reaction (PCR) and gene sequencing, with a CT value of 16.19. The patient was admitted to the isolation unit. He received two doses of the Moderna vaccine in October 2021. According to the World Health Organization WHO classification of severity, the patient was clinically stable on room air, not requiring oxygen; he was classified as mild COVID-19 pneumonia [10]. The initial complete blood count was remarkable for leukopenia and neutropenia, along with prior thrombocytopenia (Table 1). Chest x-rays (figure 1) and high-resolution CT were done, with no new findings suggestive of a new infection and slight regression of multiple areas of consolidation, which was present before. With persistent neutropenia, the patient was started on filgrastim 300 mcg. The next day after administration, his WBCs count responded to therapy (table 1). The patient has maintained on filgrastim every 72 hours and received three more doses. The patient was discharged from the isolation facility after three weeks of admission when his antigen test for COVID-19 turned negative. He was in a good clinical condition.

Case 2

**Figure 2:**
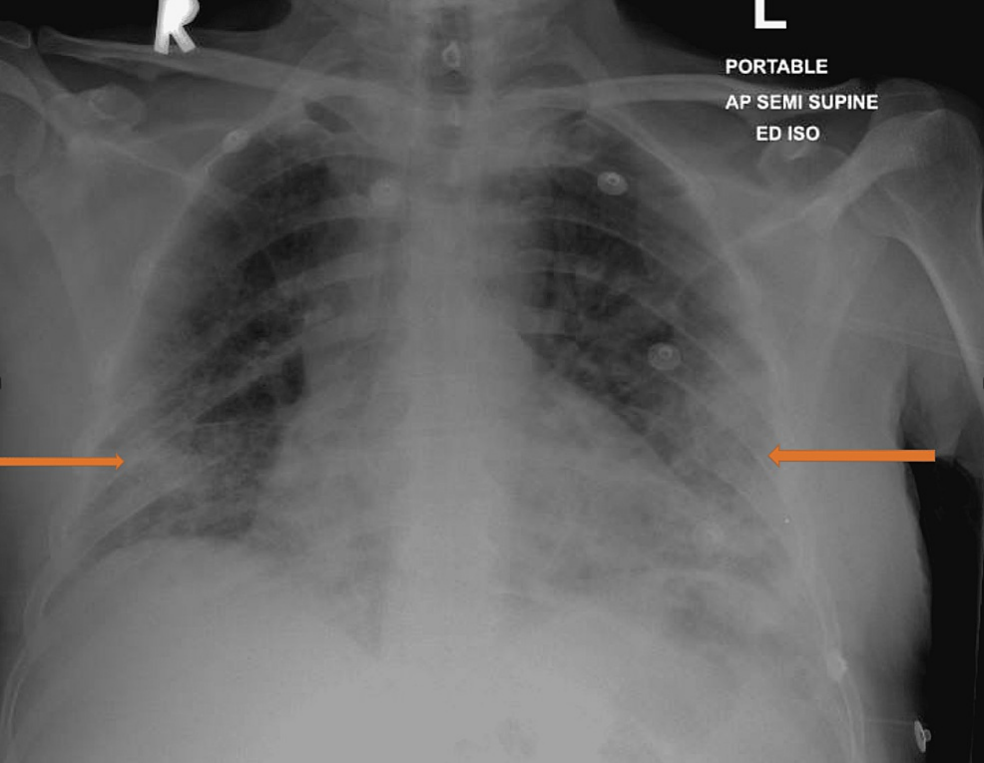
Anteroposterior chest X-ray of case 2 showing multiple airspace inhomogeneous opacities are noted on both lung fields as seen in the arrows.

A 60-year-old male with a past medical history significant only for dyslipidemia and subclinical hypothyroidism had received two doses of the Pfizer vaccine. The first dose was in February 2021, followed by one month by the second dose. In January 2022, he tested positive for the Omicron variant of SARS-CoV-2 using PCR and gene sequencing with a CT value of 16.36 and was on home isolation. One week later, while in-home isolation, he presented to the hospital with complaints of fever, cough, and shortness of breath. On admission, he was tachypneic with a respiratory rate of 28, oxygen saturation was 88% on room air, and required 10 L/min oxygen on a simple face mask. Chest x-ray showed scattered bilateral opacities (figure 2). He was admitted with severe COVID-19 pneumonia according to WHO criteria of severity [10] and started on treatment as per local protocol with remdesivir 200 mg intravenous (IV) loading dose for one day, followed by 100 mg IV daily for four days (total of five days), dexamethasone 8 mg intravenously for total 10 days, and enoxaparin 40 mg subcutaneously for prophylaxis against venous thromboembolism. After starting treatment, he improved and was off oxygen after six days. Initial complete blood counts and peripheral smear showed leukocytosis with circulating blasts (table 1). A complete blood count was done five months before admission and was expected. Bone marrow aspiration and flow cytometry confirmed the diagnosis of acute myeloid leukemia with aberrant expression of CD56. The patient stayed for about one week at the COVID-19 specialized unit and then transferred to National Center for Cancer Care and Research (NCCCR) to start his chemotherapy.

Case 3

**Figure 3:**
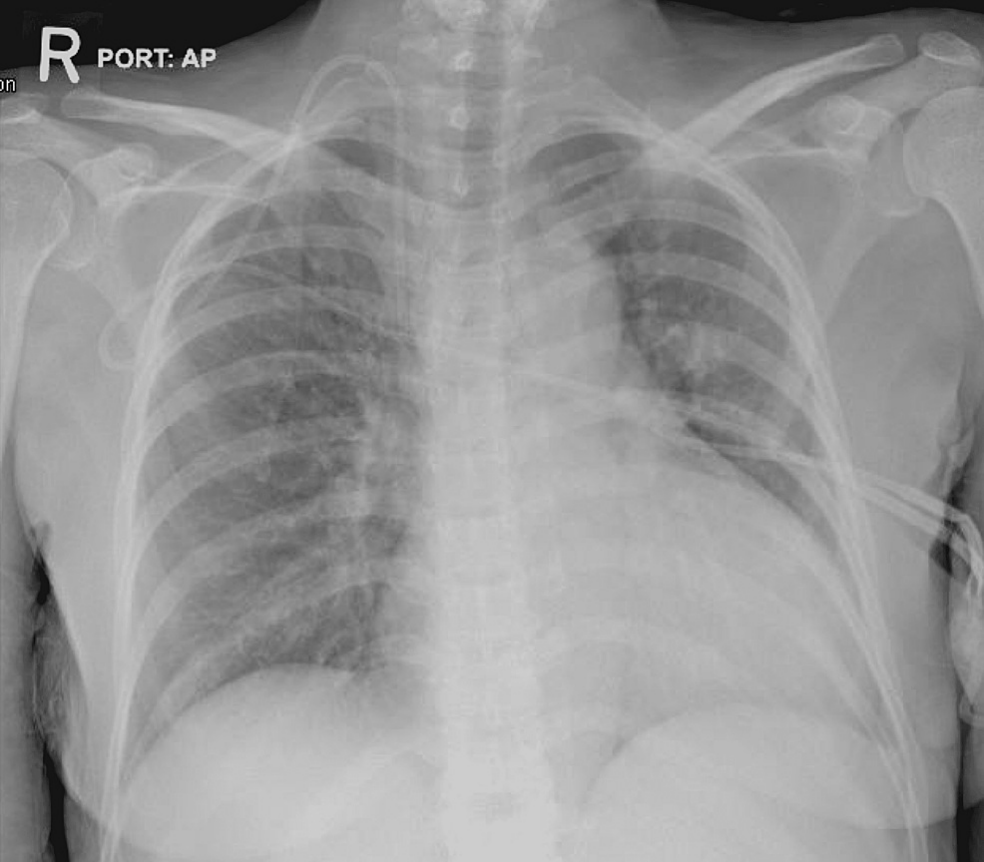
Anteroposterior chest X-ray of case 3 showing increased Broncho-vascular markings

A 52-year-old female known to have high-risk acute myeloid leukemia with increased monocytic cells received induction therapy followed by salvage FLAG-IDA therapy. He had received two doses of the Pfizer vaccine, and the first dose was in February 2021, followed by one month after the second dose. In January 2022, the patient was admitted to the cancer center (NCCCR) to start the second cycle of chemotherapy. The patient complained of cough; then, COVID-19 PCR was done, which came positive with a CT value of 16.88, and gene sequencing confirmed Omicron. A chest x-ray did not show new changes compared to previous x-rays (figure 3). According to the WHO classification of severity, the patient was on room air, not requiring oxygen, and had mild COVID-19 illness [10]. Blood investigations are shown in (table 1). He was transferred to an isolation facility and stayed there for about two weeks with no oxygen requirement. He was then transferred back to National Center for Cancer Care and Research (NCCCR) to continue her care. 

**Table 1:** Shows the laboratory finding of the three patients (cases 1, 2, and 3). WBC: white blood cell count; RBC: Red Blood Cell Count; Hgb: Hemoglobin level; Hct: Hematocrit; MCV: mean cell volume; MCH: mean corpuscular hemoglobin; RDW-CV: red cell distribution width - coefficient of variation; PDW: platelet distribution width; MPV: mean platelet volume; INR: International normalized ratio

Patient 1	Result	Reference	Patient 2	Result	Reference	Patient 3	Result	Reference
WBC	1.5 x10^3/uL	4.0-10.0	WBC	14.0 x10^3/uL	4.0-10.0	WBC	0.1 x10^3/uL	4.0-10.0
RBC	2.8 x10^6/uL	4.5-5.5	RBC	2.4 x10^6/uL	4.5-5.5	RBC	3.1 x10^6/uL	3.8-4.8
Hgb	7.7 gm/dL	13.0-17.0	Hgb	8.0 gm/dL	13.0-17.0	Hgb	8.8 gm/dL	12.0-15.0
Hct	22.90%	40.0-50.0	Hct	23.00%	40.0-50.0	Hct	25.30%	36.0-46.0
MCV	83.4 fL	83.0-101.0	MCV	96.2 fL	83.0-101.0	MCV	81.9 fL	83.0-101.0
MCH	28.2 pg	27.0-32.0	MCH	33.5 pg	27.0-32.0	MCH	28.6 pg	27.0-32.0
RDW-CV	15.30%	11.6-14.5	Platelet	51 x10^3/uL	150-400	MCHC	34.9 gm/dL	31.5-34.5
Platelet	3 x10^3/uL	150-400	MPV	10.6 fL	7.4-10.4	RDW-CV	18.10%	11.6-14.5
Absolute Neutrophil count	0.7 x10^3/uL	2.0-7.0	PDW	10.6 fL	9.4-10.6	Platelet	9 x10^3/uL	150-400
Lymphocyte count	0.3 x10^3/uL	1.0-3.0	Absolute Neutrophil count	0.5 x10^3/uL	2.0-7.0	MPV	10.7 fL	7.4-10.4
Basophil count	0.00 x10^3/uL	0.02-0.10	Lymphocyte count	6.7 x10^3/uL	1.0-3.0	Absolute Neutrophil count	0.0 x10^3/uL	2.0-7.0
Neutrophil Auto %	44.40%		Monocyte count	6.7 x10^3/uL	0.2-1.0	Lymphocyte count	0.0 x10^3/uL	1.0-3.0
Lymphocyte Auto %	21.50%		Eosinophil count	0.0 x10^3/uL	0.0-0.5	Monocyte count	0.0 x10^3/uL	0.2-1.0
Monocyte Auto %	33.80%		Basophil Auto %	0.00%		Eosinophil count	0.0 x10^3/uL	0.0-0.5
Eosinophil Auto %	0.10%		Urea	8.8 mmol/L	2.5-7.8	Basophil count	0.00 x10^3/uL	0.02-0.10
Basophil Auto %	0.20%		Creatinine	94 umol/L	62-106	Neutrophil Auto %	29.00%	
urea	2.2 mmol/L	2.5-7.8	Sodium	141 mmol/L	133-146	Lymphocyte Auto %	41.10%	
Creatinine	57 umol/L	62-106	Potassium	3.8 mmol/L	3.5-5.3	Monocyte Auto %	28.00%	
Sodium	135 mmol/L	133-146	Chloride	109 mmol/L	95-108	Urea	3.9 mmol/L	2.5-7.8
Potassium	3.9 mmol/L	3.5-5.3	Bicarbonate	20 mmol/L	22-29	Creatinine	49 umol/L	44-80
Chloride	101 mmol/L	95-108	Calcium	1.94 mmol/L		Sodium	131 mmol/L	133-146
Bicarbonate	22 mmol/L	22-29	Adjusted Calcium	2.36 mmol/L	2.20-2.60	Potassium	3.9 mmol/L	3.5-5.3
Calcium	2.23 mmol/L		Phosphorus	0.98 mmol/L	0.80-1.50	Chloride	98 mmol/L	95-108
Adjusted Calcium	2.39 mmol/L	2.20-2.60	Magnesium	0.79 mmol/L	0.70-1.00	Bicarbonate	19 mmol/L	22-29
Total Protein	68 gm/L	60-80	Total Protein	63 gm/L	60-80	Phosphorus	1.28 mmol/L	0.80-1.50
Albumin Lvl	32 gm/L	35-50	Albumin Lvl	19 gm/L	35-50	Magnesium	0.77 mmol/L	0.70-1.00
Uric Acid	214 umol/L	200-430	Uric Acid	340 umol/L	200-430	Bilirubin T	6 umol/L	0-21
Alk Phos	105 U/L	40-129	Alk Phos	43 U/L	40-129	Total Protein	63 gm/dL	60-80
ALT	84 U/L	0-41	ALT	46 U/L	0-41	Albumin Lvl	28 gm/L	35-50
AST	32 U/L	0-40	AST	39 U/L	0-40	Alk Phos	119 U/L	35-104
LDH	124 U/L	135-225	LDH	326 U/L	135-225	ALT	9 U/L	0-33
Glu Random	7.2 mmol/L		CRP	315.1 mg/L	0.0-5.0	AST	7 U/L	0-32
CRP	70.3 mg/L	0.0-5.0	D-Dimer	87.94 mg/L FEU	0.00-0.49	CRP	123.4 mg/L	0.0-5.0
INR	1.2		Ferritin	2,519.0 ug/L	30.0-553.0	INR	1.1	
CT value	16.19	< 30 indicates high viral load	INR	1		CT value	16.88	< 30 indicates high viral load
			CT value	16.88	< 30 indicates high viral load			

## Discussion

SARS COV-2 is a respiratory virus that causes the highest number of infections worldwide. Recently, many variants of the SARS-COV2 virus have emerged. The new variants resulted in pandemic waves of the COVID-19, to which people are less protected by vaccination than the wild virus (alpha variant). Omicron (B.1.1.529 lineage) was first reported from Botswana and South Africa in November 2021. The emergence of the Omicron variant resulted in a colossal pandemic wave that involved millions of people. Compared to other variants, Omicron is characterized by a high replication rate compared to different strains [11], ability to escape the humoral immune response, high rate of reinfection [12], and lower severity and hospitalization [13]. Most of the reports showed that Omicron causes milder disease. Interestingly, in patients with chronic myeloid leukemia, the Omicron infection was reported to cause mild disease [14]. However, the effect on patients with acute leukemia is not well known.

The data regarding COVID-19 infection in acute leukemia patients is scarce, mainly on the wild (alpha) variant. Data showed that patients with acute leukemia reported having an increased risk of morbidity and mortality [9,15]. Our reported cases showed that acute leukemia patients could present with mild illness and have severe disease with the Omicron variant, as seen in case 2. Of the three patients, two were known patients of acute leukemia, and one patient was newly diagnosed after the SARS COV-2 infection. Two patients had mild COVID-19 illness and did not require oxygen therapy. The three patients had low CT values (16.19, 16.35, and 16.88 for patients 1,2 and 3, respectively), indicating a high rate of viral replication. Additionally, they turned negative after three weeks, two weeks, and one week, slightly prolonged than the average duration of shedding in immunocompetent patients, 7-10 days. 

It is difficult to estimate the degree of protection vaccines provide in preventing the Omicron variant from causing the disease or reducing its severity as most of the population is vaccinated, including leukemia patients. Additionally, the number of vaccine doses required to achieve the aimed protection is unclear. Moreover, patients with leukemia are immunocompromised, and the vaccine's effectiveness in this group of patients is expected to be lower than in other populations [16]. Our reported patients had two doses, and one of them had severe disease. However, the effect on patients who were not immunized or had not encountered the virus before is unclear. It is unknown if unvaccinated patients with leukemia will develop severe respiratory symptoms, although previous immunization has little effect on Omicron infection. Among the reported cases, the only patient with severe disease was the second patient newly diagnosed with acute leukemia. The other two patients had mild illnesses. It is not clear if treatment with leukemia will protect against severe disease and which stage is more sensitive to the SARS COV-2 infection. This patient had severe covid pneumonia and immediately after recovery from covid, was started on treatment for leukemia, and he did well. This supports the finding in the Istanbul study [15] that delay in treatment does not always result in disease progression.

## Conclusions

According to these reported cases, the Omicron version of SARS COV-2 can cause mild illness in patients with acute leukemia and severe disease. This appears to be different in non-leukemic patients, where Omicron is usually softer and does not necessitate hospitalization. The challenge of treating or not treating acute leukemia during the active infection is complex, and a multidisciplinary team decision to individualize the care is needed until more concrete data support the right approach. A large study is required to fully comprehend the Omicron effect in patients with acute leukemia and its treatment.
